# Effectiveness of image‐guided radiotherapy for locally advanced esophageal squamous cell carcinoma patients treated with definitive concurrent chemoradiotherapy

**DOI:** 10.1111/1759-7714.13244

**Published:** 2019-11-19

**Authors:** Yao‐Hung Kuo, Hsin‐Yuan Fang, Yu‐Sen Lin, Ming‐Yu Lein, Chi‐Ying Yang, Shih‐Chi Ho, Chia‐Chin Li, Chun‐Ru Chien

**Affiliations:** ^1^ Department of Radiation Oncology, E‐Da Hospital Kaohsiung, Taiwan; College of Medicine, I‐Shou University Kaohsiung Taiwan; ^2^ Department of Chest Surgery China Medical University Hospital Taichung Taiwan; ^3^ School of Medicine, College of Medicine China Medical University Taichung Taiwan; ^4^ Division of Hematology and Oncology, Department of Internal Medicine China Medical University Hospital Taichung Taiwan; ^5^ Division of Hepatogastroenterology, Department of Internal Medicine China Medical University Hospital Taichung Taiwan; ^6^ Department of Radiation Oncology China Medical University Hospital Taichung Taiwan; ^7^ Department of Radiation Oncology China Medical University Hsinchu Hospital Hsinchu Taiwan

**Keywords:** Concurrent chemoradiotherapy, esophageal squamous cell carcinoma, image‐guided radiotherapy

## Abstract

**Background:**

Image‐guided radiotherapy (IGRT) is an advanced radiotherapy technique to improve the accuracy of treatment delivery. However, a recent randomized controlled trial (RCT) for prostate cancer patients treated with radiotherapy either via IGRT or routine care (no daily IGRT) reported a statistically significant worse overall survival for those treated with IGRT. This raised the concern regarding the effectiveness of IGRT for definitive concurrent chemoradiotherapy (dCCRT) for locally advanced esophageal squamous cell carcinoma (LA‐ESqCC).

**Methods:**

Eligible LA‐ESqCC patients diagnosed between 2011 and 2015 were identified via the Taiwan Cancer Registry. We estimated propensity scores to construct a 1:1 propensity‐score‐matched groups and balance observable potential confounders. The hazard ratio (HR) of death as well as other outcomes was compared between IGRT and non‐IGRT matched groups during the entire follow‐up period. The impact of additional covariables was considered in the sensitivity analysis.

**Results:**

Our study population included 590 patients in the primary analysis. The HR for death when IGRT was compared with non‐IGRT was 0.92 (95% confidence interval 0.77–1.10, *P* = 0.35). There were also no significant differences for other outcomes or sensitivity analyses.

**Conclusions:**

In this updated nonrandomized study using real world data, we found that the overall survival of LA‐ESqCC patients treated with dCCRT was not statistically different between those treated with IGRT versus those without IGRT, although the hazard ratio was less than unity, ie, in favor of IGRT. The results should be interpreted with caution given the nonrandomized design and RCTs are needed to clarify our findings.

**Key points:**

- Significant findings of the study: The OS of LA‐ESqCC patients treated with dCCRT was not statistically different between those treated with IGRT versus those without IGRT, although the hazard ratio was less than unity, ie, in favor of IGRT.
- What this study adds: In this updated nonrandomized study using real world data with additional potential confounders, our study provided a reasonable tentative evidence of lack of RCT as suggested in the literature.

## Introduction

Esophageal cancer is one of the leading causes of cancer‐related mortality worldwide.^1^ Although adenocarcinoma is one of the predominant histological subtypes in the western world, squamous cell carcinoma is most prevalent in Asia.^1^, ^2^

For locally advanced esophageal squamous cell carcinoma (LA‐ESqCC), definitive concurrent chemoradiotherapy (dCCRT) is one of the most common treatment strategies.^3^, ^4^, ^5^

Image‐guided radiotherapy (IGRT) is an advanced form of ancillary radiotherapy technique advocated in reviews and textbooks.^6^, ^7^, ^8^ IGRT employs imaging (usually x‐rays) before and/or during the delivery of radiotherapy to improve the precision and accuracy of treatment delivery.^7^ Conceptually, the improvement of radiotherapy delivery accuracy may lead to improved outcome, as reported in our previous nonrandomized study utilizing the Taiwan Cancer Registry (TCR).^9^

However, a recent randomized controlled trial (RCT) in 2018 by de Crevoisier *et al*. for prostate cancer patients treated with radiotherapy via either IGRT or routine care (no daily IGRT) reported a statistically significantly worse overall survival for those treated with IGRT, although side‐effects and disease control were improved.^10^ This raised the concern regarding the effectiveness of IGRT for other scenarios such as dCCRT for LA‐ESqCC.

Since 2011, additional prognostic factors, such as body mass index (BMI), use of alcohol, betel nuts or smoking, and use of positron emission tomography (PET), were prospectively collected in the TCR. Because these potential confounders were not adjusted in our previous study due to data limitation at that time, we aimed to investigate the effectiveness of IGRT for LA‐ESqCC patients treated with dCCRT in this updated analysis with consideration of the above potential confounders.

## Methods

### Data source

In our retrospective cohort study, the data is derived from the Health and Welfare Data Science Center (HWDC) database including the Taiwan Cancer Registry (TCR), death registration and reimbursement data for the whole population of Taiwan provided by the Bureau of National Health Insurance (NHI). All the above data were included in the HWDC with personal identifiers removed. The TCR is a high‐quality database that provides complete information such as patient/disease/treatment characteristics and prognostic factor details.^11^ This study was approved by the Research Ethics Committee, National Health Research Institutes (CMUH104‐REC‐003).

### Study population and study design

**Figure 1:**
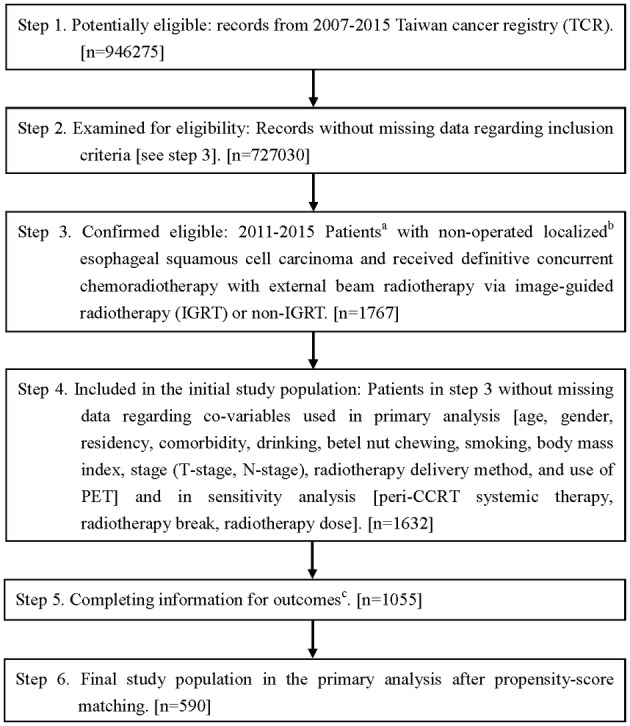
STROBE study flowchart and the number of individuals at each stage of the study. ^a^We only included those treated (class 1–2) by any single institution to ensure data consistency. ^b^The Seventh American Joint Committee on Cancer staging clinical stage II–III. ^c^Without missing information in the TCR and death registry regarding survival status, cause of death, and clinical response.

The main study flow chart designed to conform to the STROBE statement^12^ is depicted in Fig 1. The study population consisted of nonoperated locally advanced esophageal squamous cell carcinoma (LA‐ESqCC) adult (age > = 18) patients diagnosed from 2011–2015 who received concurrent systemic therapy and external beam radiotherapy 50–70 Gy using conventional fractionation via image‐guided radiotherapy (IGRT) or non‐IGRT. We excluded patients with other cancer(s) and determined the explanatory variable of interest (IGRT vs. non‐IGRT), the primary outcome of interest (overall survival [OS]) and other supplementary outcomes (complete clinical response [cCR], incidence of esophageal cancer mortality [IECM], other cancer mortality [IOCM] and cardiovascular mortality [ICVM]) based on the TCR or determined via the death registry. The date of diagnosis in the TCR was defined as the index date and OS was calculated from the date of diagnosis to the date of death or 31 December 2017 (censoring date of the death registry). The related covariables were collected based on our experiences in clinical care and TCR studies^9^, ^13^ to adjust for potential nonrandomized treatment selection (see below). We estimated the propensity scores (PS) via the above covariables to construct a PS‐matched sample and then evaluated the effectiveness of IGRT.

### Other explanatory covariables

Patient demographics (age, gender, residency), patient characteristics (comorbidity, drinking, betel nut chewing, smoking, body mass index [BMI]), disease characteristics (T‐stage, N‐stage), treatment characteristics (radiotherapy [RT] delivery), and prognostic factor (use of PET) were included in the primary analysis. Three “variables of ambiguous status,” which were “perhaps slightly affected by the treatment, but plausibly standing in as a surrogate for an important covariable that was not measured” were included in the sensitivity analysis (SA)^14^: the use of peri‐CCRT (ie, induction or consolidative) systemic therapy, radiotherapy break, and radiotherapy dose (see Statistical and Sensitivity Analysis). The covariables were defined as follows. Age was classified as at least 65 years old or not. Patient residency region was classified as northern Taiwan or elsewhere. Comorbidity was classified as with or without via Carlson comorbidity score. The drinking, betel nut chewing, smoking, and use of PET covariables were classified as yes or no. Clinical stage was classified as T1–T2 versus. T3–T4 for T‐stage and negative versus positive for N‐stage. RT delivery was classified as 3D conformal radiotherapy (3DCRT) or intensity‐modulated radiotherapy (IMRT). Peri‐CCRT systemic therapy was classified as with or without. RT break was defined as more than one week or not.

### Statistical and sensitivity analysis

In the primary analysis (PA), we used the propensity‐score (PS) method as advocated in the literature to balance the measured potential confounders.^15^, ^16^, ^17^, ^18^ We evaluate the probability of receiving IGRT (vs. non‐IGRT) via a logistic regression model based on all the above covariables, and then used the logit of the probability as the PS. The standardized difference (SDif) was used to assess the balance of covariates between 1:1 PS‐matched groups.^19^, ^20^ We compared the hazard ratio (HR) of death between IGRT and non‐IGRT matched groups during the entire follow‐up period via a robust variance estimator.^16^ As suggested in the recent literature,^21^ we also used the E‐factor to evaluate the impact of potential unmeasured confounding factor(s) on OS. Binary outcomes (cCR) within the matched pairs were compared using McNemar's test. We adopted the subdistribution HR via the clustered Fine‐Gray model to evaluate IECM, IOCM and ICVM.^22^ In the sensitivity analysis (SA), we reanalyzed what we did in the PA when we considered an additional three covariables (peri‐CCRT systemic therapy, RT break, and RT dose).

We performed the statistical analyses using the software SAS 9.4 (SAS Institute, Cary, NC) and R (R Development Core Team, R Foundation for Statistical Computing, Vienna, Austria) version 3.5.3.

## Results

### Identification of the study population used in the primary analysis

As shown in Fig 1, the identified initial study population consisted of 1632 nonoperated esophageal LA‐ESqCC cancer adult patients who received IGRT or non‐IGRT in 2011–2015. A total of 590 eligible PS‐matched patients were used as our primary study population and divided into two groups (IGRT group [*n* = 295] vs. non‐IGRT group [*n* = 295]). Radiotherapy was predominantly delivered via IMRT. The patient characteristics are described in Table 1. All covariables after PS‐matching were well balanced with small standardized differences (<0.1) although some could not be well balanced before PS‐matching.

**Table 1:** Patient characteristics of the study population in the primary analysis

		Unmatched population					Matched study population				
		IGRT (*n* = 297)		non‐IGRT (*n* = 758)			IGRT (*n* = 295)		non‐IGRT (*n* = 295)		
		Number or mean (sd)†	(%)†	Number or mean (sd)†	(%)†	SDif†	Number or mean (sd)†	(%)†	Number or mean (sd)†	(%)†	SDif†
Age	< 65	219	(74)	590	(78)	0.096	219	(74)	222	(75)	0.023
	≥ 65	78	(26)	168	(22)		76	(26)	73	(25)	
Gender	Female	13	(4)	45	(6)	0.071	13	(4)	12	(4)	0.017
	Male	284	(96)	713	(94)		282	(96)	283	(96)	
Residency	Non‐north	214	(72)	518	(68)	0.081	212	(72)	217	(74)	0.038
	North	83	(28)	240	(32)		83	(28)	78	(26)	
Comorbidity	Without	179	(60)	466	(61)	0.025	178	(60)	178	(60)	0
	With‡	118	(40)	292	(39)		117	(40)	117	(40)	
T‐stage	T1‐T2	51	(17)	83	(11)	0.180	49	(17)	45	(15)	0.037
	T3‐T4	246	(83)	675	(89)		246	(83)	250	(85)	
N‐stage	Negative	19	(6)	82	(11)	0.158	19	(6)	18	(6)	0.014
	Positive	278	(94)	676	(89)		276	(94)	277	(94)	
RT delivery	3DCRT	§	§	§	§	0.241	§	§	§	§	0
	IMRT	§	§	§	§		§	§	§	§	
Use of PET	No	69	(23)	198	(26)	0.067	69	(23)	67	(23)	0.016
	Yes	228	(77)	560	(74)		226	(77)	228	(77)	
Drinking	No	42	(14)	112	(15)	0.018	41	(14)	40	(14)	0.010
	Yes	255	(86)	646	(85)		254	(86)	255	(86)	
Betel nut chewing	No	151	(51)	335	(44)	0.133	149	(51)	148	(50)	0.007
	Yes	146	(49)	423	(56)		146	(49)	147	(50)	
Smoking	No	44	(15)	99	(13)	0.051	43	(15)	42	(14)	0.010
	Yes	253	(85)	659	(87)		252	(85)	253	(86)	
BMI		22.01 (3.36)		21.47 (3.61)		0.154	22.00 (3.37)		22.06 (3.82)		0.016

† Rounded.

‡ Carlson comorbidity score ≥ 1.

§ The exact numbers were not reported because of a Health and Welfare Data Science Center (HWDC) database center policy to avoid numbers in single cells (≤2).

3DCRT, 3D conformal radiotherapy; BMI, body mass index; IGRT, image‐guided radiotherapy; IMRT, intensity‐modulated radiotherapy; PET, positron emission tomography; RT, radiotherapy; sd, standard deviation; SDif, standardized difference.

### Primary analysis

**Figure 2:**
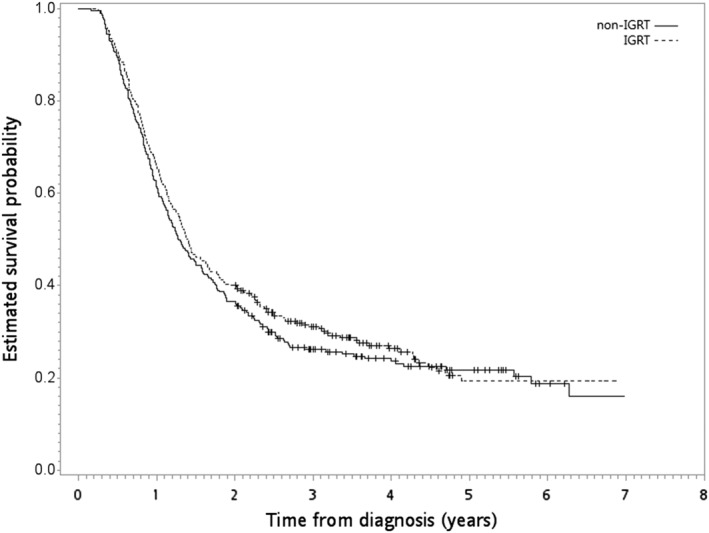
Kaplan‐Meier overall survival curve (in years) in the primary analysis. (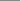) non‐IGRT and (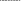) IGRT.

After a median follow‐up of 16 months (range 2–84 months), death was observed for 218 patients in the IGRT group and for 227 in the non‐IGRT group. The HR of death when IGRT was compared to non‐IGRT was 0.92 (95% confidence interval [95% CI] 0.77–1.10, *P* = 0.35). The observed HR 0.92 for OS could be explained by an unmeasured confounder associated with the selection of treatment (IGRT or non‐IGRT) and survival by a risk ratio of 1.31 (E‐value) fold each, but weaker confounding factors could not do so. The five‐year OS rates for IGRT versus non‐IGRT were 19% and 22%. Figure 2 shows the Kaplan‐Meier survival curve for OS. The results of the HR for IECM (HR = 0.92, *P* = 0.37), IOCM (HR = 1.1, *P* = 0.8) and ICVM (HR = 1, *P* = 1) were also not significantly different. The cCR rates were 24% versus 22% for IGRT versus non‐IGRT groups (*P* = 0.63).

### Sensitivity analysis (SA)

When considering an additional three covariables (peri‐CCRT systemic therapy, RT break, and RT dose), we were still able to construct balanced study populations after PS‐matching (Table 2). The HR for death when IGRT was compared with non‐IGRT was similar as in the PA (HR = 0.93; 95% CI 0.77–1.12; *P* value = 0.42). The five‐year OS rates for IGRT versus non‐IGRT were 19% and 20%. There was also no statistically significant difference for the results of the other outcomes (HR = 0.95, *P* = 0.62 for IECM; HR = 0.99, *P* = 0.98 for IOCM; HR = 0.66, *P* = 0.65 for ICVM) and the distribution of the cCR rates (24% vs. 27% for IGRT vs. non‐IGRT groups, *P* = 0.41].

**Table 2:** Patient characteristics of the study population in the sensitivity analysis

		IGRT (*n* = 295)		non‐IGRT (*n* = 295)		
		Number or mean (sd)†	(%)†	Number or mean (sd)†	(%)†	SDif†
Age	< 65	219	(74)	223	(76)	0.031
	≥ 65	76	(26)	72	(24)	
Gender	Female	13	(4)	9	(3)	0.072
	Male	282	(96)	286	(97)	
Residency	Non‐north	212	(72)	209	(71)	0.022
	North	83	(28)	86	(29)	
Comorbidity	Without	178	(60)	178	(60)	0
	With‡	117	(40)	117	(40)	
T‐stage	T1‐T2	49	(17)	47	(16)	0.018
	T3‐T4	246	(83)	248	(84)	
N‐stage	Negative	19	(6)	16	(5)	0.043
	Positive	276	(94)	279	(95)	
RT delivery	3DCRT	§	§	§	§	0
	IMRT	§	§	§	§	
Use of PET	No	69	(23)	63	(21)	0.049
	Yes	226	(77)	232	(79)	
Drinking	No	41	(14)	37	(13)	0.040
	Yes	254	(86)	258	(87)	
Betel nut chewing	No	149	(51)	147	(50)	0.014
	Yes	146	(49)	148	(50)	
Smoking	No	43	(15)	41	(14)	0.019
	Yes	252	(85)	254	(86)	
BMI		22.00 (3.37)		21.97 (3.69)		0.007
Peri‐CCRT systemic therapy	Without	206	(70)	207	(70)	0.007
	With	89	(30)	88	(30)	
RT break	≤1 week	205	(69)	209	(71)	0.030
	>1 week	90	(31)	86	(29)	
RT dose (Gy)		57.75 (6.67)		57.91 (6.54)		0.024

† Rounded.

‡ Carlson comorbidity score ≥ 1.

§ The exact numbers were not reported because of a Health and Welfare Data Science Center (HWDC) database center policy to avoid numbers in single cells (≤2).

3DCRT, 3D conformal radiotherapy; BMI, body mass index; CCRT, concurrent chemoradiotherapy; IGRT, image‐guided radiotherapy; IMRT, intensity‐modulated radiotherapy; PET, positron emission tomography; RT, radiotherapy; sd, standard deviation; SDif, standardized difference.

## Discussion

In this updated nonrandomized study using real‐world data with additional potential confounders, we found that the OS of LA‐ESqCC patients treated with dCCRT was not statistically different between those treated with IGRT versus those without IGRT, although the hazard ratio was less than unity (ie, in favor of IGRT).

From the viewpoint of evidence‐based medicine, our finding was not of the highest level of evidence^23^, ^24^ and interpretation must therefore be cautious due to the nonrandomized study design.^24^ However, when we searched the clinical trial registry (https://clinicaltrials.gov/) in August 2019 using the keywords “(image‐guided radiation therapy) OR (image‐guided radiotherapy) OR (IGRT) | Phase 2, 3, Not Applicable”, we found no RCT regarding IGRT for LA‐ESqCC patients treated with dCCRT. Therefore, our study provided reasonable tentative evidence that there is a lack of RCT as suggested in the literature.^24^

We searched for relevant studies by searching PubMed in August 2019 using the following keywords “((IGRT) OR (Image‐guided Radiation Therapy) OR ((image*) AND (guid*) AND ((radiotherapy) OR (radiation therapy)))) AND survival AND (esophageal cancer)”. We found no relevant study except our previous one^9^ and another known single arm study.^24^ In our previous study, without consideration of the additional covariables as mentioned in the Introduction, we found OS was improved by IGRT with HR 0.82 in the primary analysis but the results were not robust in sensitivity analyses. When additional “variables of ambiguous status” were considered in the propensity score model, the HR was 0.95 (*P* = 0.5). In the present study, the HR was insignificantly in favor of IGRT in both the primary or sensitivity analysis. The inclusion of the additional covariables only available in the current study may at least partly explain the differences between our previous and the current study. However, the observed HR for death was less than unity (ie, still in favor of IGRT), in contrast to the HR 2.12 [*P* = 0.042] observed in the RCT for prostate cancer.^10^

Our updated analysis was inspired by the unexpected results in the RCT for prostate cancer^10^ which deserved further discussion. Please note that overall survival was the secondary but not the primary outcome of the prostate RCT, in which recurrence‐free survival (primary outcome) was not different between the groups. Furthermore, higher incidence of other cancer (HR 2.28) and cardiovascular mortality (6/236 vs. 1/234) was observed in the post‐hoc analyses of this prostate study, which were all possible radiotherapy sequelae. However, the interpretation of secondary outcomes and post‐hoc analysis should be cautious but not conclusive, and similar incidences of other cancer mortality and cardiovascular mortality were observed in our study.

There were several limitations in this study. First, potential unmeasured confounders are always a limitation of a nonrandomized study as seen previously^9^ and the current study in which the treatment selection was not randomized and the reason for IGRT (vs. non‐IGRT) could not be ascertained in TCR. Although we had included additional covariables in the current study, there were still possible unmeasured confounders. For example, taxane has been used in modern RCT with excellent results,^25^ but this systemic therapy detail was not available in our study due to data limitation. Also, the detail of planning target volume (PTV) margin was not available in TCR. In addition, accessibility (IGRT may not be available in some institutes) or treatment era (more recent patients may have had a greater chance to be treated with IGRT) issues may be possible because IGRT is a relatively recent technology, although the item “IGRT” was coded in TCR since the establishment of modern TCR in year 2007. So patients treated with IGRT may have been those who had access to more modern technology and therefore a better prognosis. Therefore, we reported E‐value to quantify the potential impact of unmeasured confounder(s) as suggested in the literature. Second, the impact of novel modalities such as carbon ions^26^ or immune therapy^27^ was not considered in this study. Third, other endpoints such as recurrence‐free survival as used in the prostate cancer IGRT trial^10^ or side effects might be more relevant to the effect of IGRT. However, these endpoints could not be reliably obtained from our data sets to our knowledge. Finally, the intervention (IGRT) in our study was not homogenous. To our knowledge, several different forms of IGRT are available in Taiwan, including, but not limited to, cone beam computed tomography, kV imaging, and mV imaging, which could not be differentiated in the TCR. However, it is unclear whether these various technologies lead to different clinical benefits or not.^6^

In conclusion, in this updated nonrandomized study using real‐world data with additional potential confounders, we found that the OS of LA‐ESqCC patients treated with dCCRT was not statistically different between those treated with IGRT versus those without IGRT, although the hazard ratio was less than unity (ie, in favor of IGRT). The results should be interpreted with caution given the nonrandomized design of the study. RCTs are needed to clarify this finding.

## Disclosure

The authors have not published or submitted the manuscript elsewhere. The authors declare no conflict of interest.
